# What is the true *in vitro* potency of oxytetracycline for the pig pneumonia pathogens *Actinobacillus pleuropneumoniae* and *Pasteurella multocida*?

**DOI:** 10.1111/jvp.12386

**Published:** 2017-01-18

**Authors:** L. Dorey, S. Hobson, P. Lees

**Affiliations:** ^1^ Department of Comparative Biological Sciences The Royal Veterinary College Hatfield Herts UK; ^2^ Norbrook Laboratories Ltd. Newry Co. Down UK

## Abstract

The pharmacodynamics of oxytetracycline was determined for pig respiratory tract pathogens, *Actinobacillus pleuropneumoniae* and *Pasteurella multocida*. Indices of potency were determined for the following: (i) two matrices, broth and pig serum; (ii) five overlapping sets of twofold dilutions; and (iii) a high strength starting culture. For *A. pleuropneumoniae,* minimum inhibitory concentration (MIC) was similar for the two matrices, but for *P. multocida,* differences were marked and significantly different. MIC and minimum bactericidal concentration (MBC) serum: broth ratios for *A. pleuropneumoniae* were 0.83:1 and 1.22:1, respectively, and corresponding values for *P. multocida* were 22.0:1 and 7.34:1. For mutant prevention concentration (MPC) serum: broth ratios were 0.79:1 (*A. pleuropneumoniae*) and 20.9:1 (*P. multocida*). These ratios were corrected for serum protein binding to yield fraction unbound (fu) serum: broth MIC ratios of 0.24:1 (*A. pleuropneumoniae*) and 6.30:1 (*P. multocida*). Corresponding fu serum: broth ratios for MPC were almost identical, 0.23:1 and 6.08:1. These corrections for protein binding did not account for potency differences between serum and broth for either species; based on fu serum MICs, potency in serum was approximately fourfold greater than predicted for *A. pleuropneumoniae* and sixfold smaller than predicted for *P. multocida*. For both broth and serum and both bacterial species, MICs were also dependent on initial inoculum strength. The killing action of oxytetracycline had the characteristics of codependency for both *A. pleuropneumoniae* and *P. multocida* in both growth media. The *in vitro* potency of oxytetracycline in pig serum is likely to be closer to the *in vivo* plasma/serum concentration required for efficacy than potency estimated in broths.

## Introduction

The tetracycline group of antibiotics, which includes oxytetracycline and doxycycline, has consistently had the highest veterinary sales volume in the United Kingdom of the seven antimicrobial drug classes analysed by Veterinary Antimicrobial Resistance and Sales Surveillance (UK‐VARSS). They possess a broad spectrum of activity. However, resistance to drugs of this group is common. For example, de Jong *et al*. (2014) reported that, for pig isolates, 22% of *Pasteurella multocida*, 15% of *Actinobacillus pleuropneumoniae* and 82% of *Streptococcus suis* were classified as resistant to tetracycline.

Oxytetracycline has been in extensive veterinary use, for more than 60 years, in farm animal medicine. Although usually classified as a bacteriostat, *in vitro* studies have demonstrated bactericidal actions for pneumonia pathogens (Brentnall *et al*., 2012; Lees *et al*., 2016). In this *in vitro* study, antimicrobial potency and efficacy of oxytetracycline against two pathogenic species, implicated in pig pneumonia, *P. multocida* and *A. pleuropneumoniae,* were investigated. The indices measured were as follows: (i) minimum inhibitory concentration (MIC), the lowest *in vitro* concentration which inhibits visible growth; (ii) minimum bactericidal concentration (MBC), the concentration which produces a 3 log_10_ reduction in bacterial count; (iii) mutant prevention concentration (MPC), the concentration preventing the growth of the least susceptible cells in high‐density bacterial populations (Blondeau *et al*., 2004); and (iv) time–kill curves quantifying the time course of growth inhibition.

The European Union Committee on Antimicrobial Sensitivity Testing (EUCAST) and the Clinical Laboratory Standards Institute (CLSI) have defined the internationally accepted methods, standards and guidelines for MIC and MBC determinations. Such standardization is essential when comparing data from several laboratories, between countries and across time periods. Moreover, it establishes cut‐off values for antimicrobial sensitivity testing (Papich, 2013). These bodies require twofold dilutions for MIC determinations, and this is important because, when plotted on a histogram using a log‐base 2 distribution, the distributions are log‐normal. These histograms are therefore readily examined for the purpose of identifying wild‐type distributions. The CLSI and EUCAST standards thus enable determination of ECOFF and cut‐offs for wild‐type organisms.

For the purposes of this study, the standardized CLSI/EUCAST methods of determining MIC and MBC have two disadvantages. As they are based on twofold dilutions, there is potential for up to 100% error on single isolate estimates, thus having a limitation regarding accuracy for each isolate. From pharmacological and therapeutic dose determination perspectives, it is accuracy and not precision that is the key objective. To meet, in part, this concern on accuracy, previous studies in this laboratory have used five sets of overlapping twofold dilutions to reduce inaccuracy from approaching 100% to not more than 20% (Aliabadi & Lees, 2001; Sidhu *et al*., 2010). Secondly, the internationally recommended CLSI/EUCAST standards are based on the use of artificial growth media, such as cation‐adjusted Mueller–Hinton broth (CAMHB). Whilst these provide optimal growth conditions *in vitro*, they differ in composition (chemically and immunologically) from biological fluids and therefore may not be representative of bacterial growth conditions *in vivo*. To provide comparisons between broth and biological fluid growth matrices, and to establish possible differences between them, previous MIC, MBC and time–kill studies in our laboratory have been undertaken in calf serum and inflammatory exudate (Aliabadi & Lees, 2001, 2002; Sidhu *et al*., 2010).

For some drug classes, differences between artificial broths and biological fluids for determining antimicrobial drug potency and efficacy are small, and unlikely to have a major impact on dose determination, provided the nonprotein bound serum drug concentration is used, as the protein bound fraction is microbiologically inactive (Gonzalez *et al*., 2013; Toutain *et al*., 2016). For other drug classes, differences between broths and biological fluids may be large. Brentnall *et al*. (2012) reported for a calf isolate of *Mannheimia haemolytica* an oxytetracycline MIC in serum 19 times *greater* than the broth MIC. In contrast, Illambas *et al*. (2009) and Toutain *et al*. (2016) reported MICs some 50‐fold *smaller* in calf serum compared to broth for tulathromycin for *M. haemolytica* and *P. multocida* calf isolates. Hence, the quantitative determination of pharmacodynamic indices with improved accuracy and in biological fluid matrices should be regarded, for some drug classes, as appropriate and even necessary for determination of PK/PD break points and the application of PK/PD principles to dose determination. Furthermore, Zeitlinger *et al*. (2004) commented ‘investigations without determination of protein binding must be considered highly speculative…..bacteria with appropriate and well‐defined growth in the selected medium should be employed….in order to be able to extrapolate data from various models to *in vivo* situations, models should always attempt to mimic physiological conditions as closely as possible’.

A third consideration, in respect of optimizing clinical efficacy and minimizing the emergence of resistance to antimicrobial drugs, is dosage in relation to pathogen load. For prophylaxis, metaphylaxis and treatment early in the course of disease, when the pathogen load is low or absent, many drugs will, at recommended dosages, either prevent or cure disease, in concert with natural body defences. The major challenge, however, is to achieve a bacteriological cure when pathogen density in the biophase is high. For this reason, in this study, a high starting inoculum count of approximately 10^7 ^CFU/mL was selected, in preference to the inoculum count of 5 × 10^5 ^CFU/mL recommended in CLSI and EUCAST guidelines for MIC and related studies.

The aims of this investigation were as follows: (i) to determine the degree of protein binding of oxytetracycline in pig serum; (ii) for oxytetracycline against six isolates each of *A. pleuropneumoniae* and *P. multocida* (known respiratory pathogens in the pig) (a) to determine MICs using five sets of overlapping twofold dilutions, (b) to compare MICs, MBCs and MPCs of oxytetracycline in two matrices, artificial broth and pig serum, (c) to establish time–kill curves in both growth matrices for eight multiples of MIC, using high starting inoculum counts; (iii) for two isolates each of *P. multocida* and *A. pleuropneumoniae* to investigate the effect on MIC of (a) varying the initial inoculum pH over the range 7.0–8.0, (b) increasing the calcium and magnesium concentrations of CAMHB; and (iv) for six isolates each of *P. multocida* and *A. pleuropneumoniae* to determine MICs using low, intermediate and high starting inoculum counts.

## Materials and Methods

### Serum protein binding of oxytetracycline

A high‐pressure liquid chromatography (HPLC) method with ultraviolet (UV) detection was used to quantify oxytetracycline concentrations in pig serum (Horspool & McKellar, 1990). The HPLC system comprised a Dionex Ultimate 3000 pump and autosampler, connected to a Dionex UVD340S detector (Thermo Fisher UK Ltd., Boundary Way, Hemel Hempstead, UK). UV detection was set to wavelength 354 nm, and the retention time of oxytetracycline was 4 min. Chromatographic data were analysed using Chromeleon version 6.80, and concentrations of oxytetracycline were calculated using ratios of peak area, compared to the internal standard. Recovery of the drug was determined by comparing the peak area of standards in methanol and standards in serum, when known oxytetracycline concentrations were spiked into methanol and serum, respectively. The spiking concentrations were 0, 0.1, 0.25, 0.5, 1, 2.5, 5, 10 and 25 μg/mL.

Two sample sets were used as follows: (i) spiked oxytetracycline standards in serum to provide the total concentration of protein bound and free drug and (ii) serum samples prepared as in (i) then filtered using ultrafiltration devices (Amicon Ultra Centrifugal filters, Ultracel 10 k, Sigma‐Aldrich Company Ltd., Dorset, UK). Briefly, a 3‐mL aliquot was placed in the ultrafilter unit and centrifuged at 4000 ***g*** for 20 min at 25 °C. The ultrafiltrate was assayed for drug concentration to estimate oxytetracycline concentration in the protein free fraction. For each concentration, unbound concentration was determined for three serum samples.

### Origin, storage and selection of bacterial isolates

Twenty isolates of *P. multocida* were supplied by Don Whitley Scientific, Shipley, West Yorkshire, UK. They also supplied three ATCC reference strains, *A. pleuropneumoniae* ATCC 27090, *Enterococcus faecalis* ATCC 29212 and *Escherichia coli* ATCC 25922 to validate MIC determinations. Eight isolates of *A. pleuropneumoniae* were supplied by A. Rycroft (Royal Veterinary College, Hawkshead Campus, Hatfield, Herts., UK). All *P. multocida* and *A. pleuropneumoniae* isolates were derived from EU field cases of pig pneumonia. They were stored in 10% Marvel^®^ milk powder, 15% glycerol and sterile distilled H_2_O to 100% at −80 °C. The mixture was sterilized by boiling for 5 sec, allowed to cool for 12 h and then boiled again for a further 5 sec.

Based on three criteria, six isolates of each species were selected: (i) ability to grow logarithmically in both broth and pig serum, (ii) susceptibility to oxytetracycline in disc diffusion assays (data not shown), and (iii) the highest and lowest broth MICs and four isolates with intermediate MICs, determined using twofold dilutions (data not shown). This initial selection procedure ensured that all isolates could be used in subsequent investigations in both growth media and that they comprised a small but diverse range of susceptible isolates.

### Culture methods and bacterial counts

For *A. pleuropneumoniae,* chocolate Mueller–Hinton agar (CMHA) was used to grow the organism on a solid medium and Columbia broth supplemented with 2 μg/mL nicotinamide adenine dinucleotide (NAD) was used as the liquid broth. CLSI guidelines require use of Veterinary Fastidious Medium for the liquid culture of *A. pleuropneumoniae*. However, for isolates used in this study, MIC end point was more readily and reliably established using Columbia broth. Mueller–Hinton agar supplemented with 5% defibrinated sheep blood (MHA) was used to grow *P. multocida*. The liquid medium used for *P. multocida* was CAMHB. They were incubated in a static incubator at 37 °C for 18–24 h.

Bacterial counts were determined by serial dilution and spot plate counts. Tenfold or 100‐fold dilutions were made in phosphate‐buffered saline. Three 10 μL drops of the appropriate dilutions were dropped onto the agar surface and allowed to dry for 10 min prior to incubating. After 24‐h incubation, the mean colony‐forming unit (CFU) count for each 10 μL was determined and multiplied by 100 to give the CFU/mL and then multiplied by the dilution factor to obtain the starting count.

### Minimum inhibitory and minimum bactericidal concentrations

MICs and MBCs were determined by microdilution for six isolates each of *A. pleuropneumoniae* and *P. multocida,* based on CLSI guidelines (2008) and using CLSI recommended broth (for *P. multocida* but not for *A. pleuropneumoniae*). In addition, MICs were determined in 100% pig serum. For both growth media, five overlapping sets of twofold serial dilutions of oxytetracycline were prepared, with a large initial range from lowest set of 0.0078 to 128 μg/mL and highest set of 0.014 to 230 μg/mL in 96‐well plates. Bacterial cultures were grown in broth or serum to 0.5 McFarland Standard, corresponding to approximately 1–2 × 10^8 ^CFU/mL. This was diluted tenfold to obtain the intended starting inoculum, 1–2 × 10^7^ CFU/mL, which is higher than CLSI recommendation of 5 × 10^5 ^CFU/mL. The higher inoculum count was deliberately selected, to be equivalent to a medium to heavy clinical challenge and to facilitate comparison with *in vitro* time–kill curves (*vide infra*).

Oxytetracycline solutions, media and bacterial cultures were added to each cell based on the concentration/volume formula, C1V1 = C2V2; that is for a concentration of 2 μg/mL oxytetracycline, the following were added to the well: 4 μL of 100 μg/mL oxytetracycline stock solution added to 194 μL of broth or serum and then 2 μL of culture. The plate was sealed and incubated statically for 24 h at 37 °C. Spot plate counts were prepared immediately after plate inoculation.

Each test with each isolate was repeated in triplicate for confirmation and validation. Growth was indicated by turbidity or appearance of a growth spot, and the MIC was the lowest concentration which inhibited visible growth. ATCC reference strains (*vide supra*) were used in all assays, but the starting counts were 1.10^5 ^CFU/ml, as required by CLSI methods. The positive control well contained medium and pathogen and was expected to yield a positive growth result; the negative control well contained medium and oxytetracycline and was expected to yield no growth; and the blank control contained medium only and was expected to achieve no growth. If results were not within the expected range, the test was repeated.

MBCs were determined in accordance with CLSI standards (2008). The 96 wells were‐examined for growth to determine the MIC and, in addition to that well, wells containing five subsequent concentrations higher than MIC were established by spot plating. This indicated a 3 log_10_ reduction in inoculum count.

### Mutant prevention concentration

Fresh cultures were grown on agar, and approximately 100 single CFUs were collected, with a sterile dampened swab and used to inoculate culture from plates into a volumetric flask containing 200 mL of prewarmed broth. This was placed in a static incubator overnight at 37 °C. The next day, 1 mL of culture was added to 9 mL broth and placed in an orbital incubator at 37 °C and 180 rpm for 4 h. After 4 h, the bacterial suspension yields a theoretical count of 1–2 × 10^11 ^CFU/mL. A spot plate was used to confirm inoculum density. For *P. multocida*, as a rapidly growing pathogen*,* no centrifuge step was required. However, for the more fastidious organism, *A. pleuropneumoniae,* a centrifuge step was used when required. The drug concentrations were 1, 2, 4, 8, 16, 32, 64 and 128 multiples of the MIC for each isolate. The concentration ranges were narrowed down two further times; for example, if MPC was 64× MIC, the next range would be 32, 36, 40, 44, 48, 52, 56, 60 and 64× MIC, and the final range if the MPC was 36× MIC would be 32, 32.5, 33, 33.5, 34, 34.5, 35, 35.5 and 36× MIC.

Oxytetracycline solution (0.5 mL) was applied to cold, dry agar plates and left to dry; then, 100 μL of culture was added to the plate and allowed to dry. Plates were incubated at 37 °C for 72 h and checked for growth every 24 h. MPC was recorded as the lowest oxytetracycline concentration that inhibited bacterial growth completely after 72‐h incubation. The method was validated against that described by Blondeau (2009). This provided full confidence in the current methodology. For each of six isolates of each organism, triplicate measurements were carried out, using both broth and serum as growth matrices.

### Antimicrobial growth (time–kill) curves

Two to three colonies of the test isolate were added to 5 mL broth or serum and incubated overnight in an orbital incubator under standard conditions, as described above. Fifty microlitres of this culture was diluted 1:50 in prewarmed broth or serum and incubated statically for 1 h at 37 °C to a 0.5 McFarland Standard. This was then diluted tenfold to obtain the intended starting inoculum, to be consistent with MIC determinations at 1–2 × 10^7 ^CFU/mL, and confirmed using colony counts. Drug concentrations of eight multiples of MIC (0.25, 0.5, 1, 1.5, 2, 4, 6 and 8× MIC) were prepared in prewarmed broth or serum. Ten microlitres of the prepared culture was used to inoculate the dilutions to yield a 1 mL final volume. Fifty microlitres of each culture was sampled, and the count was determined by serial dilution and spot plates at nine times: 0, 0.25, 0.5, 0.75, 1, 2, 4, 8 and 24‐h incubation. The negative control contained no organism, whilst for the positive control, no drug was added. Spot plates were examined for contamination. Each test was repeated in triplicate for six isolates of both organisms grown in both broth and serum. The lower limit of quantification (LLOQ) was 33 CFU/mL.

### Monitoring pH before and after MIC determination

The pH of serum was regularly monitored in all experiments, as, after 24‐h incubation, pH may change with the release of products of bacterial metabolism. To quantify pH change from start to end of incubation in MIC studies, the macrodilution method was used. The MIC determinations were made in triplicate in both broth and serum for two isolates each of *P. multocida* and *A. pleuropneumoniae*.

### Effect of pH on MIC

The initial pH of each medium, broth and serum, was adjusted to values of 7.0, 7.2, 7.4, 7.6, 7.8 or 8.0 using hydrochloric acid (HCl, 1 m) or sodium hydroxide (NaOH, 1 m) MICs were determined by macrodilution and repeated in triplicate with five overlapping sets of twofold dilutions as a preliminary study for two isolates each of *P. multocida* and *A. pleuropneumoniae*.

### Effect of cation adjustments on MIC

CLSI guidelines suggest the use of CAMHB for the culture of *P. multocida* and the calcium and magnesium ion concentrations of CAMHB, to meet CLSI standards, are in the ranges 20–25 mg/L Ca^++^ and 10–12.5 mg/L Mg^++^. The concentrations of these divalent cations were increased by 0, 5, 10, 15, 20, 25, 30 mg/L Ca^++^ by adding CaCl_2_ stock solution to 1 L CAMHB and by 0, 3, 6, 10, 12, 15 and 18 mg/L Mg^++^ by adding 10 mg/mL MgCl_2_ stock solution to 1 L CAMHB. MIC determinations were repeated three times as a preliminary study for two *P. multocida* and *A. pleuropneumoniae* isolates. *Actinobacillus pleuropneumoniae* was investigated for comparative purposes with *P. multocida*.

### Effect of inoculum size on MIC

To determine the effect of initial inoculum size on MIC, three initial inoculum strengths were evaluated as follows: high (10^8 ^CFU/mL), intermediate (10^6 ^CFU/mL) and low (10^4 ^CFU/mL). To prepare the cultures, a 0.5 McFarland standard was made; this has a theoretical value of 1.5 × 10^8 ^CFU/mL (high). Two 1:10 dilutions provide a theoretical value of 1.5 × 10^6^ CFU/mL (intermediate). A further two 1:10 dilutions give a theoretical value of 1.5 × 10^4^ CFU/mL (low). MICs were determined in triplicate in broth and serum for six isolates each of *P. multocida* and *A. pleuropneumoniae*, using five overlapping sets of twofold dilutions.

### Data collection and analysis

Data were recorded on Microsoft Excel and processed using GraphPad Prism v6. The data were analysed using IBM SPSS Statistics 22 by Kruskal–Wallis H with *post hoc* Mann–Whitney *U*‐test.

## Results

### Serum protein binding

The standard curve for concentrations of oxytetracycline in serum was linear for eight concentrations over the range 0.1–25 μg/mL. The intra‐assay CV%s for three batches of serum were 24.1, 18.5 and 15.2%. The mean (SD) percentage protein binding for oxytetracycline in pig serum was 70.9 (9.19) and was independent of total concentration.

### Minimum inhibitory, minimum bactericidal and mutant prevention concentrations

**Figure 1:**
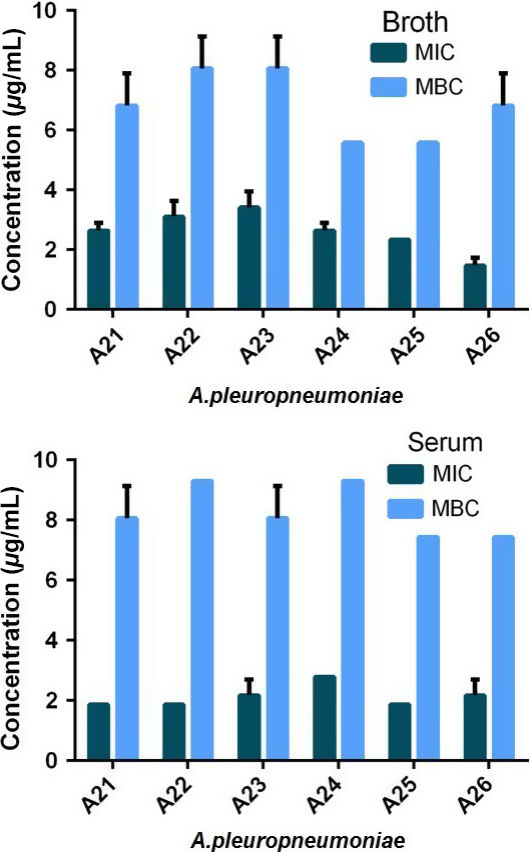
Individual isolate MICs and MBCs for oxytetracycline for *Actinobacillus pleuropneumoniae* A21‐A26 in broth (upper graph) and serum (lower graph). Standard deviation bars indicate repeatability (*n* = 3 for each isolate). [Colour figure can be viewed at wileyonlinelibrary.com].

**Figure 2:**
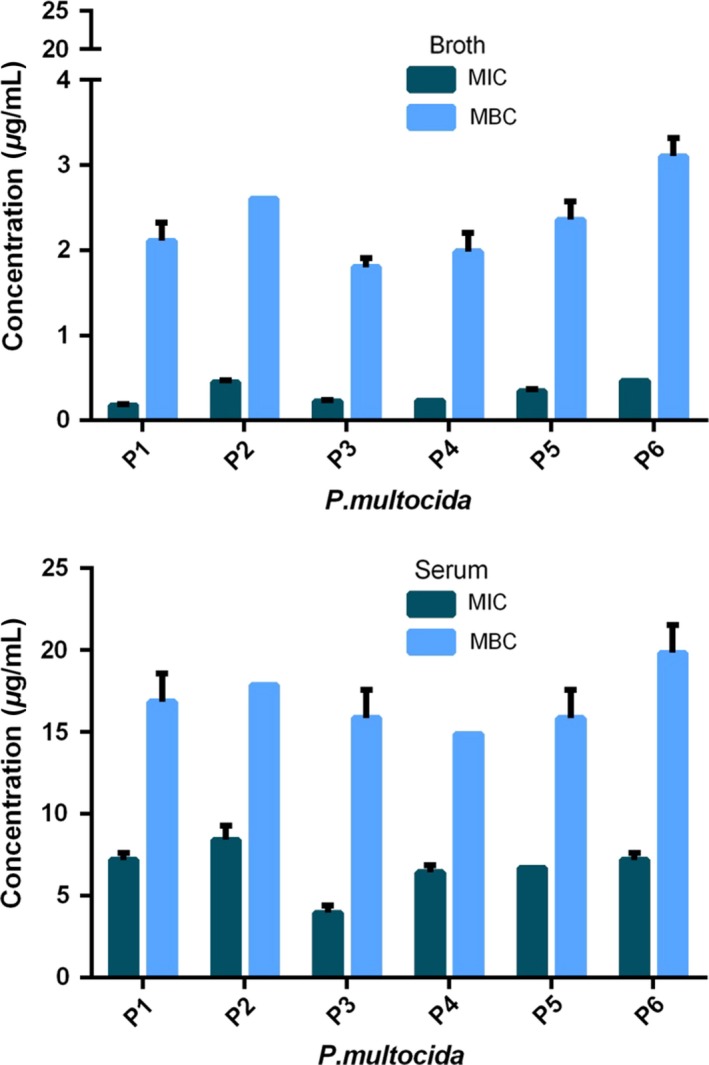
Individual isolate MICs and MBCs for oxytetracycline for *Pasteurella multocida* P1‐P6 in broth (upper graph) and serum (lower graph). Standard deviation bars indicate repeatability (*n* = 3 for each isolate). Note differing ordinate scales. [Colour figure can be viewed at wileyonlinelibrary.com].

Figures 1, 2 illustrate MIC and MBC for *A. pleuropneumoniae* and *P. multocida* in broth and serum, indicating the interisolate variability and reproducibility with triplicate analyses per isolate. Table 1 presents geometric means of MIC, MBC and MPC in broth, serum and serum MIC corrected for drug binding to serum protein, that is fraction unbound (fu) serum MIC. Table 2 indicates serum: broth and fu serum: broth ratios for each of these indices and Table 3 presents the ratios MBC: MIC and MPC: MIC for each growth matrix. For repeatability, the coefficient of variation ranged from 5 to 11% for *P. multocida* serum MIC and 2 to 24% for *A. pleuropneumoniae* serum MIC.

**Table 1:** *Actinobacillus pleuropneumoniae* (APP) and *Pasteurella multocida* (PM) MIC, MBC and MPC for broth, serum and free concentration in serum (fu serum)†

Organism	Medium	MIC	MBC	MPC
APP	Broth	2.50 (0.70)	6.71 (0.43)	44.35 (12.63)
	Serum	2.08 (0.43)	8.22 (0.95)*	35.09 (6.66)*
	fu Serum	0.61 (0.13)**	2.39 (0.28)**	10.21**
PM	Broth	0.30 (0.12)	2.28 (0.47)	6.84 (2.76)
	Serum	6.48 (1.45)**	16.75 (2.04)**	142.84 (33.27)**
	fu Serum	1.89 (0.42)**	4.87 (0.59)*	41.57**

^†^fu serum = serum concentrations corrected for protein binding. Geometric means (SD) of oxytetracycline concentrations for each of six isolates of each organism determined in triplicate. Significant differences of serum and fu serum from broth values: *P < 0.05, **P < 0.01.

**Table 2:** Serum:broth and fu serum:broth ratios of MIC, MBC and MPC for *Actinobacillus pleuropneumoniae* (APP) and *Pasteurella multocida* (PM)

Organism	Medium	MIC	MBC	MPC
APP	Serum:broth	0.83:1	1.22:1	0.79:1
	fu Serum:broth	0.24:1	0.36:1	0.23:1
PM	Serum:broth	21.96:1	7.34:1	20.89:1
	fu Serum:broth	6.30:1	2.14:1	6.08:1

fu serum = serum concentrations corrected for protein binding. Ratios for triplicate analyses for each of six isolates of each organism.

**Table 3:** MBC: MIC and MPC: MIC ratios for *Actinobacillus pleuropneumoniae* (APP) and *Pasteurella multocida* (PM) in broth and serum

Organism	Medium	MBC:MIC	MPC:MIC
APP	Broth	2.7:1	17.7:1
	Serum	4.0:1	16.8:1
PM	Broth	7.7:1	22.8:1
	Serum	2.6:1	22.0:1

Ratios for triplicate analyses for each of six isolates of each organism.

For *A. pleuropneumoniae*, MICs were similar in serum and broth; the geometric mean serum: broth ratio was 0.83:1. The corresponding ratio for MBC was 1.22:1. These differences between serum and broth were not statistically significant. However, after correcting for 71% serum protein binding, the fu serum: broth MIC ratio for *A. pleuropneumoniae* was 0.24:1, indicating an approximately fourfold difference between experimental and predicted values of MIC (*P* < 0.01).

For *P. multocida,* the uncorrected serum: broth ratios were 22.0:1 for MIC and 7.34:1 for MBC (*P* < 0.01 for both indices). The fu serum: broth ratios were lower, 6.30:1 for MIC (*P* < 0.01) and 2.14:1 for MBC (*P* < 0.05) indicating greater than sixfold and twofold differences, respectively, after correction for the microbiologically inactive protein bound fraction.

For both *A. pleuropneumoniae* and *P. multocida,* MBC: MIC ratios revealed both bacterial species and growth medium differences. For *A. pleuropneumoniae,* the MBC: MIC ratio in broth was 2.7:1 compared to 4.0:1 in serum, whereas for *P. multocida,* corresponding ratios were, conversely, 7.7:1 and 2.6:1. Thus, comparing the two species, the MBC/MIC difference was greater for *P. multocida* in broth, whereas the MBC/MIC difference was greater for *A. pleuropneumoniae* in serum. The differences in MBC: MIC ratios between broth and serum were statistically significant for both species (*P* < 0.01).

**Figure 3:**
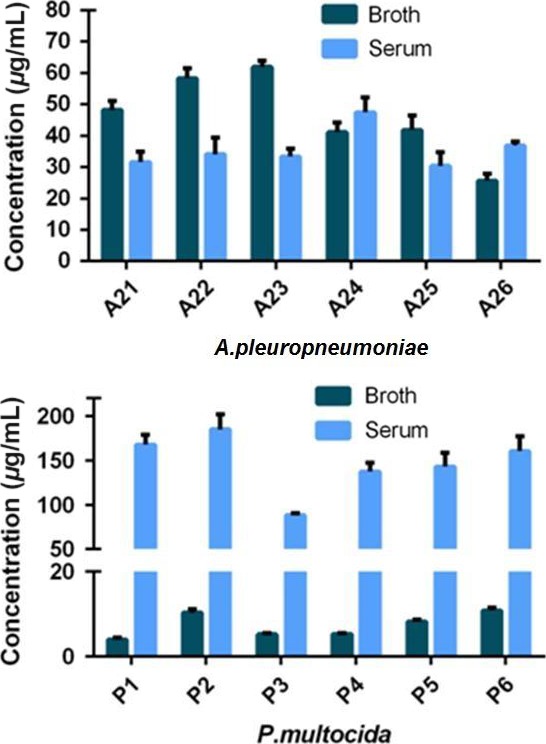
Individual isolate broth and serum MPCs for oxytetracycline for *Actinobacillus pleuropneumoniae* (upper graph) and *Pasteurella multocida* (lower graph). Standard deviation bars indicate repeatability (*n* = 3 for each isolate). Note differing ordinate scales for broth and serum for *P. multocida*. [Colour figure can be viewed at wileyonlinelibrary.com].

For the determination of MPCs, a pilot test validated the method used compared to that of Blondeau (2009) (data not shown). Geometric mean MPCs and MPC: MIC ratios for six isolates each of *A. pleuropneumoniae* and *P. multocida* are presented in Tables 1 and 3. Figure 3 illustrates the interisolate variability of MPC in broth and serum, with triplicate analysis for each isolate.

For *A. pleuropneumoniae,* MPCs exceeded MICs in both media, 17.7‐fold in broth and 16.8‐fold in serum; geometric mean MPCs were 44.4 μg/mL (broth) and 35.1 μg/mL(serum). For *P. multocida,* MPCs were 22.8× MIC in broth and 22.0× MIC in serum; geometric mean MPCs were 6.84 μg/mL (broth) and 142.8 μg/mL (serum).

The MPC serum: broth ratio for *A. pleuropneumoniae* was 0.79:1, which was similar to the MIC serum: broth ratio of 0.83:1. The corresponding MPC serum: broth ratio for *P. multocida* was 20.9:1, which was almost identical to the serum: broth MIC ratio of 22.0:1. After correcting serum MPCs for protein binding, the fu serum: broth MPC ratios were approximately fourfold *lower* (*A. pleuropneumoniae*) and sixfold *higher* (*P. multocida*) than the predicted ratio of 1:1. These differences were virtually identical to those obtained for MIC.

### pH measurement before and after MIC determinations

The pH of broth increased from 7.10 to 7.28 and in serum the pH increased from 7.27 and 7.40.

### Effect of pH on MIC

**Figure 4:**
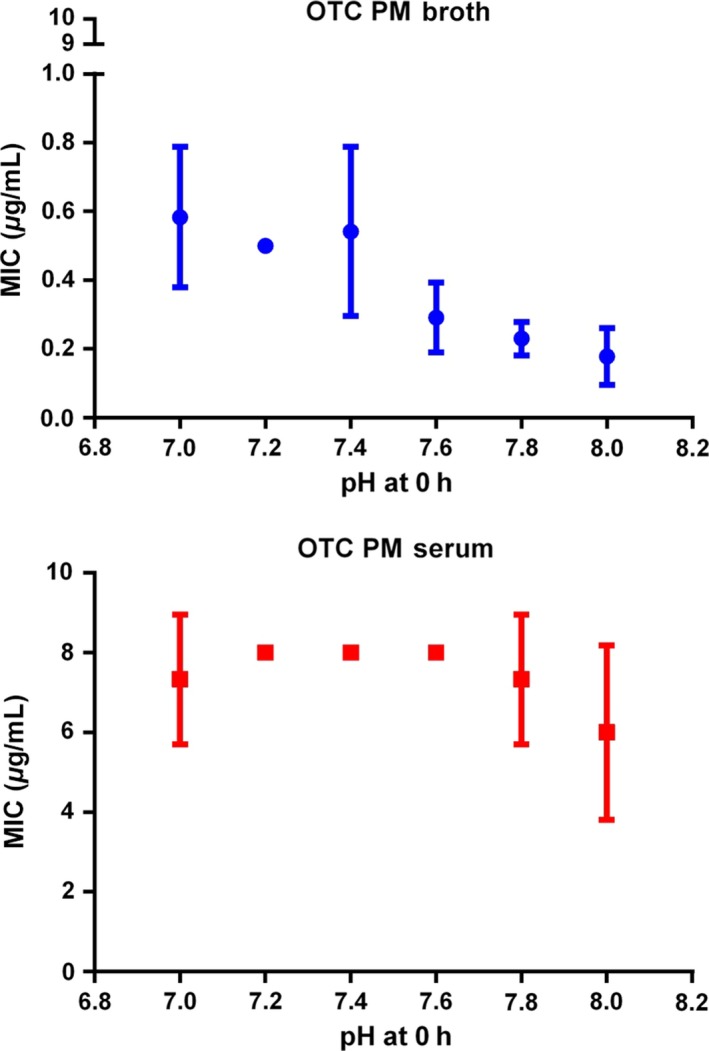
*Pasteurella multocida* MICs for two isolates in broth and serum at six initial pH values: lines of best fit and means. Standard deviation bars indicate repeatability (*n* = 3) for each isolate). Note differing scales. [Colour figure can be viewed at wileyonlinelibrary.com].

**Figure 5:**
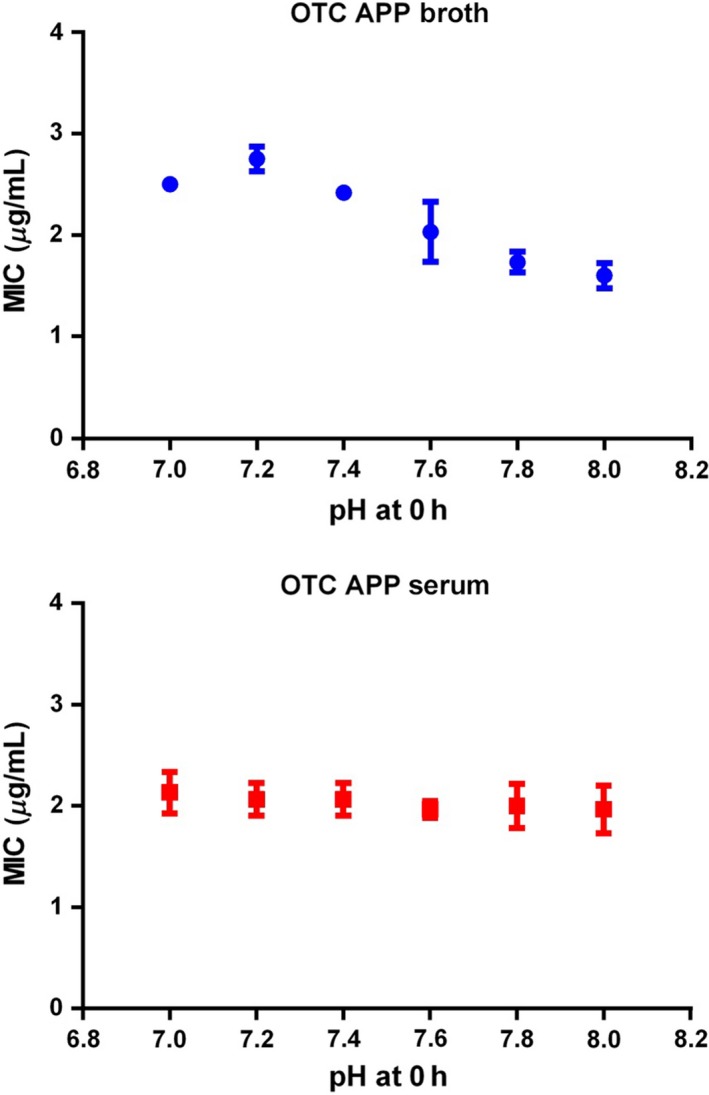
*Actinobacillus pleuropneumoniae* MICs for two isolates in broth and serum at six initial pH values: lines of best fit and means. Standard deviation bars indicate repeatability (*n* = 3) for each isolate). [Colour figure can be viewed at wileyonlinelibrary.com].

The pH of the initial culture media (broth and serum) was adjusted to 7.0, 7.2, 7.4, 7.6, 7.8 or 8.0. For both organisms, broth MICs decreased (reflecting a potency increase) progressively as pH became more alkaline, from pH 7.4 to 8.0. For serum, MICs for both species were not significantly affected by pH (Figs 4 & 5).

### Effect of cation adjustments on MIC

**Figure 6:**
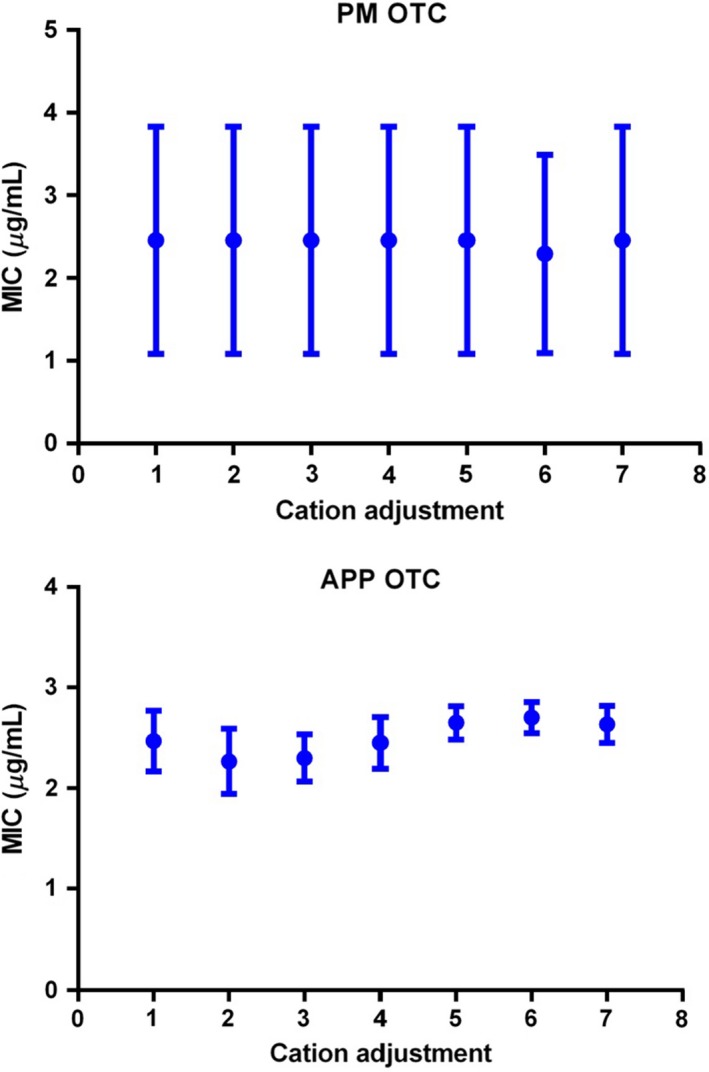
Mean and standard deviation broth (CAMHB) MICs (μg/mL) (for triplicate determinations) for two isolates of *Pasteurella multocida*. Concentrations of calcium and magnesium were increased by additions to MHB as follows: Group 1, 0 mg Ca^++^/L and 0 mg Mg^++^/L; Group 2, 5 mg Ca^++^/L and 3 mg Mg^++^/L; Group 3, 10 mg Ca^++^/L and 6 mg Mg^++^/L; Group 4, 15 mg Ca^++^/L and 10 mg Mg^++^/L; Group 5, 20 mg Ca^++^/L and 12 mg Mg^++^/L; Group 6, 25 mg Ca^++^/L and 15 mg Mg^++^/L; and Group 7, 30 mg Ca^++^/L and 18 mg Mg^++^/L. [Colour figure can be viewed at wileyonlinelibrary.com].

Concentrations of the divalent cations, calcium and magnesium in broth were increased by 0, 5, 10, 15, 20, 25, 30 mg Ca^++^/L and 0, 3, 6, 10, 12, 15 and 18 mg Mg^++^/L. These alterations to calcium and magnesium concentrations did not affect MIC for two isolates of *P. multocida* or *A. pleuropneumoniae* (Fig. 6).

### Effect of inoculum size on MIC

MICs were determined in broth and serum for six isolates each of *P. multocida* (Table 4) and *A. pleuropneumoniae* (Table 5) with three initial inoculum strengths. MICs were inoculum size dependent, increasing progressively with inoculum size. MIC ratios high: low inocula were significant (*P* < 0.01) for broth and serum for both organisms. Ratios high: medium inocula were also significant for both media (*P* < 0.05). For *P. multocida* serum: broth, MIC ratios were in the range 30:1 to 40:1 and the fu serum: broth MIC range was 8.7:1 to 11.6:1. For *A. pleuropneumoniae,* serum: broth ratios were in the range 1.03:1 to 1.41:1 and ratios of fu serum: broth were in the range 0.30:1 to 0.41:1.

**Table 4:** *Pasteurella multocida* MICs for broth and serum with three initial inoculum strengths†, ratios of MICs and serum:broth ratios

Inoculum size	Broth MIC	Serum MIC	Serum:broth ratio
High	0.82 (0.22)	28.80 (0.00)	34.91:1
Medium	0.18 (0.01)	7.23 (0.00)	40.16:1
Low	0.14 (0.02)	4.14 (0.27)	30.06:1
High:medium ratio	4.6:1*	4.0:1*	
High:low ratio	6.0:1**	7.0:1**	
Medium:low ratio	1.3:1	1.7:1	

^†^High (108 CFU/mL), medium (106 CFU/mL) and low (104 CFU/mL). Geometric mean (SD) MICs: triplicate analyses for each of six isolates. Significance of differences for high: medium, high: low and medium: low inocula: *P < 0.05, **P < 0.01.

**Table 5:** *Actinobacillus pleuropneumoniae* (APP) MICs for broth and serum with three initial inoculum strengths†, ratios of MICs and serum:broth ratios

Inoculum size	Broth MIC	Serum MIC	Serum: Broth ratio
High	0.89 (0.22)	1.26 (0.00)	1.41:1
Medium	0.27 (0.16)	0.28 (0.08)	1.02:1
Low	0.13 (0.01)	0.14 (0.01)	1.03:1
High:medium ratio	3.3:1	4.5:1*	
High:low ratio	6.7:1**	9.2:1**	
Medium:low ratio	2.1:1	2.0:1	

^†^High (108 CFU/mL), medium (106 CFU/mL) and low (104 CFU/mL). Geometric mean (SD) MICs: triplicate analyses for each of six isolates. Significance of differences for high: medium, high: low and medium: low inocula: *P < 0.05, **P < 0.01.

### Time–kill curves

**Figure 7:**
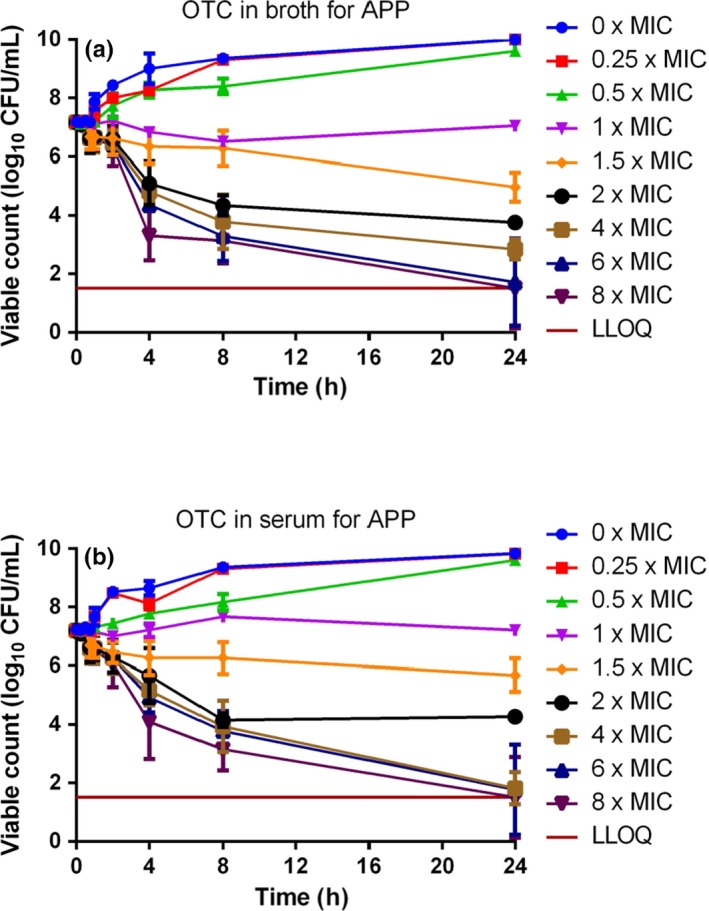
*In vitro* inhibition of growth of *Actinobacillus pleuropneumoniae* over 24‐h exposure to 8 multiples of MIC for oxytetracycline in (A) Columbia broth supplemented with NAD and (B) serum (*n* = 6). Standard deviation bars shown. LLOQ was 33 CFU/mL. [Colour figure can be viewed at wileyonlinelibrary.com].

Time–kill curves for oxytetracycline are illustrated in Figs 7 (*A. pleuropneumoniae*) and 8 (*P. multocida*). For *A. pleuropneumoniae*, little growth inhibition occurred at concentrations <1× MIC, resulting in a 2–3 log_10_ increase in CFU/mL over 24 h at 0.25 and 0.5× MIC. At 1.5× MIC, there was a 1–2 log_10_ reduction in CFU/mL by 8 h in both media. At concentrations exceeding 4× MIC, bactericidal activity occurred by 4 h, and by 24 h, there was a 5 log_10_ CFU/mL decrease in both media.

**Figure 8:**
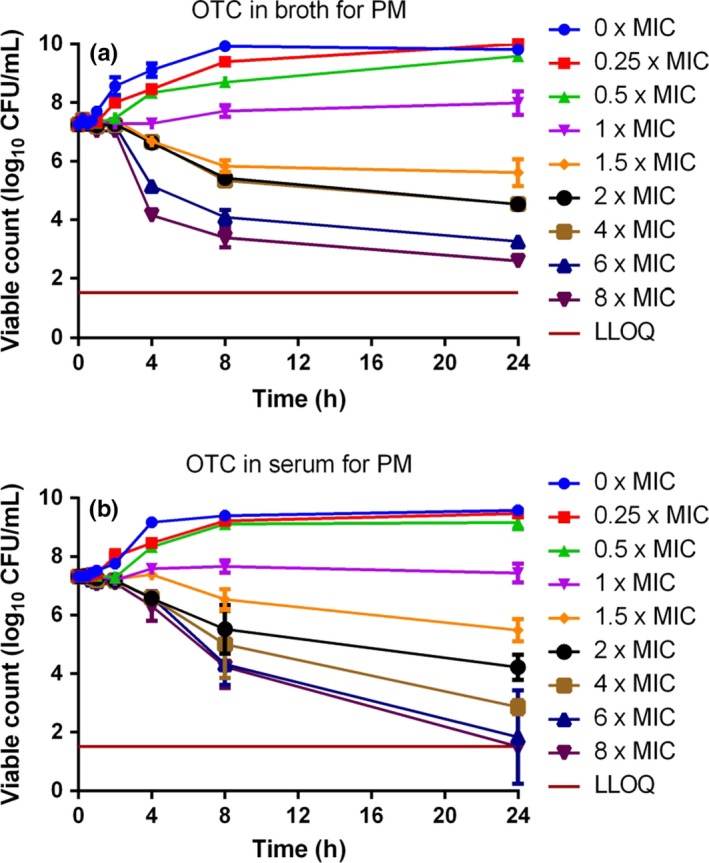
*In vitro* inhibition of growth of *Pasteurella multocida* over 24‐h exposure to 8 multiples of MIC for oxytetracycline in (A) CAMHB and (B) serum (*n* = 6). Standard deviation bars shown. LLOQ was 33 CFU/mL. [Colour figure can be viewed at wileyonlinelibrary.com].

For *P. multocida*, little growth inhibition occurred at concentrations less than MIC in both matrices. Progressively increasing inhibition was obtained at 1.5, 2 and 4× MIC in broth, and bactericidal activity occurred by 8 h at 6× MIC and 4 h at 8× MIC. In serum, for 6 and 8× MIC, a 3 log_10_ decrease in CFU/mL was obtained by 24 h. Despite a more rapid initial reduction in bacterial count in broth than in serum, by 24 h there was virtual eradication (bacterial count <33 CFU/mL) at 6 and 8× MIC in serum. The killing action of oxytetracycline was judged to be codependent in both fluids for both micro‐organisms.

## Discussion

### Indices of oxytetracycline potency and growth medium

Determination of potency for antimicrobial drugs in broths, as required by CLSI and EUCAST standards, has many advantages; their methods incorporate quality controls, break points and epidemiological/wild‐type cut‐offs, which ensure that MIC data are comparable between laboratories and across countries. This is appropriate for routine antimicrobial susceptibility testing. However, the present data suggest that serum might be a more relevant fluid than broth for potency determination, when the objective is estimation of PK/PD break points and prediction of a dose schedule for oxytetracycline for clinical use in the pig, that is based on pharmacokinetic/pharmacodynamic integration and modelling approaches.

However, CLSI/EUCAST methods of determining MIC and MBC and the Blondeau (2009) described MPC method have two disadvantages. First, being based on twofold dilutions, there is potential for up to 100% error on single isolate estimates, thus having a limitation regarding accuracy for individual isolates; this study used five sets of overlapping twofold dilutions, reducing inaccuracy to no >20%. Second, the CLSI/EUCAST standards are based on the use of growth media, such as CAMHB, formulated to facilitate growth *in vitro*. Therefore, they differ in composition from biological fluids and hence may not be fully representative of bacterial growth conditions *in vivo* (Aliabadi & Lees, 2001, 2002; Nightingale and Murakawa 2002; Zeitlinger *et al*., 2004; Zeitlinger *et al*., 2008; Sidhu *et al*., 2010).

Differences between broths and biological fluids for determining drug potency, for some drug classes, are small and may not have a significant impact on dose determination, provided the nonprotein bound plasma/serum drug concentration is known and applied. For other drug classes, however, differences between broths and biological fluids may be large. Further refinement of the methodology used in this study would be to determine potency not only in serum but also in the presence of other ‘natural’ constituents, such as leucocytes and antibodies. Moreover, the time–kill studies were based on exposure of the bacteria to fixed oxytetracycline concentrations for a predefined time period. *In vivo,* concentrations in serum and the biophase first increase and then decrease after systemic, nonvascular dosing. Therefore, *in vitro* time–kill methods, such as hollow fibre models, which better reflect the circumstances of clinical exposure, could be used in future studies to better simulate *in vivo* concentration–time profiles. Serum is a more relevant fluid than broth first for MIC determination and time–kill curves and then for PK/PD break point determination because (i) broth is nonbiological and formulated to optimize the growth of bacteria *in vitro*; (ii) for dose determination purposes, accuracy (not precision) of MIC determination is the key factor.

Variability between and within microbial species is a natural phenomenon, resulting from environmental/evolutionary influences over time. For wild‐type organisms of each species, there is corresponding interstrain variability in the antimicrobial drug potency indices, MIC, MBC and MPC for all drugs. In addition, the clinical use of antimicrobial drugs exerts selective pressures on micro‐organisms, potentially leading to reduced susceptibility to their killing actions.

It has previously been demonstrated for oxytetracycline and farm animal pathogens that growth medium can influence MIC profoundly. Brentnall *et al*. (2012) compared oxytetracycline MICs against an isolate of calf *M. haemolytica* in five matrices, reporting values of 0.5, 0.8, 14.8, 12.8 and 11.2 μg/mL in MHB, CAMHB, calf serum, calf exudate and calf transudate, respectively. A subsequent calf study from our laboratory described similar (approximately 20‐fold) MIC differences for oxytetracycline in serum compared to MHB for six calf isolates of *M. haemolytica* and *P. multocida* (Lees *et al*., 2016).

In another recent study, Honeyman *et al*. (2015) compared several tetracyclines for MICs in broth and 50% broth: 50% mouse serum. For a single strain of *Streptococcus pneumoniae,* MICs were identical for the two media for five compounds but, with added serum, twofold to fourfold increases were obtained for five, whilst MIC was increased 32‐fold for one compound. For a strain of *Staphylococcus aureus,* however, MIC was increased in the broth: serum combined matrix relative to pure broth for all 12 compounds investigated, and for seven, the increase was in the range eightfold to 128‐fold. Moreover, for the latter species, 50% human serum exerted a similar effect quantitatively as mouse serum. Therefore, as in the present investigation, the effect of serum on MIC was, for several drugs of the tetracycline group, profound and bacterial species dependent.

### Factors potentially influencing oxytetracycline potency

The serum: broth MIC ratios obtained in this study were 0.83:1 for *A. pleuropneumoniae* and 22.0:1 for *P. multocida*. Therefore, the serum/broth differences were markedly species dependent, and these differences were confirmed by similar serum: broth ratios for MBC and MPC for each species. Despite these marked matrix‐dependent differences in three inhibitory concentrations, MIC, MBC and MPC, the time–kill curves for broth and serum for each species were similar, when established using multiples of MIC. The similarity between the time–kill curves refers to the shape of the curve and consequential effect (concentration, co‐, or time‐dependent) and not of the concentrations required to elicit this effect. Therefore, we can conclude that although the drug concentration required to produce these results has the same serum: broth ratio, the shape of the curve and drug effect remains the same/similar.

The serum/broth differences in MIC, MBC and MPC were bacterial species dependent, as strikingly illustrated by the data for *P. multocida*. For this organism, correction for protein binding yielded a mean fu serum MIC of 1.89 μg/mL, whilst broth MIC for the same isolates was 0.30 μg/mL. Thus, the fu serum MIC was 6.3‐fold greater than the broth MIC, the universally accepted standard. For MPC, the difference between serum and broth after correction for protein binding was 6.1‐fold. This indicates an inhibition of the killing action of oxytetracycline by some serum factor(s). The cause requires further study by methods for example repeating the test with protein free serum.

For *P. multocida,* MIC differences between serum and broth could be due to the natural buffers or differing composition of serum compared to broth. The present findings indicate a small effect of pH on activity in broth with greater potency (reduced MIC) at alkaline pH. However, MIC was independent of pH in serum. Other investigators have reported pH dependency of broth MIC for tetracycline, penicillin, sulphonamides and aminoglycosides; the pHs for optimal activity were 6.6, 6.8, 7.3 and 7.8, respectively (Garrod & Waterworth, 1969).

For *A. pleuropneumoniae* the MIC, MBC and MPC serum:broth ratio ranges were 0.79–1.22:1, whereas for *P. multocida,* the ratio range was 7–22:1. The causes to these bacterial species differences are to be determined. Zeitlinger *et al*. (2008) compared growth curves, in the absence of antimicrobial drugs, of *S. aureus* and *Pseudomonas aeruginosa* in MHB, serum and varying proportions of serum admixed with MHB. Slower logarithmic growth was obtained for both species in serum compared to broth, and this might be expected to provide more rapid kill in serum for a given drug concentration, because of a smaller microbial challenge. Studies in our laboratory have also established slower growth rates in pig serum compared to broth for *P. multocida* and *A. pleuropneumoniae* (L. Dorey and P. Lees, unpublished data).

For *P. multocida,* the present findings, of low potency in serum relative to achievable plasma concentrations, suggest the possibility of mechanisms of action of oxytetracycline other than direct killing. The parent drug of this group, tetracycline, alters the expression of proteins in *M. haemolytica* at concentrations less than MIC (Nanduri *et al*., 2005). Altered expression of proteins involved in energy production, nucleotide metabolism and translation, and the bacterial stress response (chaperones) were reported. These authors also described a decrease in the expression of leukotoxin A, a major virulence factor of *M. haemolytica*. Another potential action of tetracyclines is on organism pathogenicity, for example by inhibition of bacterial adherence to cells (Forestier *et al*., 1984; Gabler *et al*., 1992) including epithelial lining cells in the respiratory tract (Tylewska *et al*., 1981; Lantz *et al*., 1987; Haig, 2011).

In addition to these several possible actions of tetracyclines on bacteria, modification of several functions of mammalian cells has been reported (Griffin *et al*., 2011). These include inhibition of cytokine release (Shapira *et al*., 1998), immunomodulation and anti‐inflammatory actions (Woo *et al*., 1999; Pasquale & Tan, 2005), scavenging of reactive oxygen moieties (Griffin *et al*., 2011), induction of nitric oxide synthases (Amin *et al*., 1996), inhibition of proliferation, migration and phagocytosis and also induction of apoptosis by macrophages and antiprotease activity (Fife *et al*., 1997; Hoeben *et al*., 1997; Bettany & Wolowacz, 1998; Griffin *et al*., 2011). Hence, the clinical response to oxytetracycline might involve many factors. These include the following: bacterial species and strain MIC and MBC values; inhibitory effects on bacteria at sub‐MIC concentrations; effects on organism pathogenicity; disease severity and pathogen load; early therapeutic intervention; host immunocompetence and interaction of oxytetracycline with one or several of these factors.

The influence of growth medium on MIC, MBC and MPC may be attributable to several factors, including protein binding in serum and other differences in media composition. The degree of protein binding of oxytetracycline in pig serum was 71%, and it should be noted that protein bound antimicrobial drugs are antimicrobiologically inactive (Merrikin *et al*., 1983; Wise, 1986; Zeitlinger *et al*., 2004). This value is similar to the 75.5% binding for oxytetracycline reported in piglets (Mevius *et al*., 1986) and 72% reported in cattle (Mitchell *et al*., 2012). In other cattle studies, however, lower protein binding of oxytetracycline of 53, 50 and 18.6% has been reported, respectively, by Lees *et al*. (2015), Pilloud (1973) and Ziv and Sulman (1972).

For *A. pleuropneumoniae,* when correction was made for 71% protein binding, the fu serum MIC was 4.1‐fold lower than the broth MIC. Therefore, the fu serum:broth ratio of 0.24:1 was significantly less than the 1:1 ratio anticipated on the assumption (made by most investigators) that there are no differences between serum and broth, once correction for serum protein binding has been made. Taking broth MIC as the universally accepted standard (and noting that in this study it is based on five overlapping sets of twofold dilutions), our data indicate that there are serum factor(s) which synergize with oxytetracycline to enhance its killing action against *A. pleuropneumoniae*.

In confirmation of the serum/broth differences in growth inhibition for *A. pleuropneumoniae*, it should be noted that the ‘synergism factor’ for MPC was 4.3, which is almost identical to the 4.1 factor for MIC. The factor for MBC was somewhat lower at 2.8. Enhancement of the action of oxytetracycline might simply be due to a slower rate of logarithmic growth of *A. pleuropneumoniae* in serum compared to broth (Dorey *et al*. to be published). However, elucidation of the mechanism(s) will require further study, possibly by comparison against other slow growing organisms including *Mycoplasma* spp.

For six bovine isolates of *M. haemolytica* and *P. multocida,* MIC CV% values of 84 and 38%, respectively, were reported for oxytetracycline (Lees *et al*., 2016). Similarly the CV% of *P. multocida* was small in this study which was 41% for broth MICs and 21% for serum. For *A. pleuropneumoniae,* small interisolate variability of MICs with a CV% 12% for broth and 8% for serum.

In time–kill studies, the killing action of oxytetracycline was classified as codependent in both broth and serum for both *A. pleuropneumoniae* and *P. multocida*. A conclusion of codependency was reached in other studies for *P. multocida* and *M. haemolytica* isolates harvested from calves (Brentnall *et al*., 2012; Lees *et al*., 2016). However, other authors have proposed that oxytetracycline action is time‐dependent (Mitchell *et al*., 2012; Rigos & Smith, 2015).

In this study, a high inoculum count was selected for use in the time–kill studies. The starting count (approximately 10^7 ^CFU/mL) is higher than that recommended by CLSI (2008) of 5 × 10^5 ^CFU/mL and was selected specifically to provide a major challenge to the killing action of oxytetracycline. Moreover, our additional data indicate significant increases in MIC between low and high and between medium and high starting inoculum counts; the findings therefore anticipate, unsurprisingly, a requirement for higher dosages for heavy infections in clinical use for both pathogens. The quantitative differences obtained in this study will provide a basis for calculating differential doses for differing pathogen loads and for each pathogenic species.

In the light of the potency indices of oxytetracycline in pig serum for *A. pleuropneumoniae* and *P. multocida*, and from knowledge of plasma concentrations achieved with clinical dosages (Mevius *et al*., 1986), it is clear that (i) concentrations exceeding the MPC will not be achieved *in vivo* for either pathogen; (ii) microbiologically effective concentrations will probably be obtained (for a duration to be determined and depending on pathogen load) for *A. pleuropneumoniae* but possibly not for *P. multocida. *These considerations are further reviewed in a companion paper to the present article (Dorey *et al*., 2017).

## Conclusions

In summary, matrix‐dependent effects on quantitative MIC, MBC and MPC values were obtained for oxytetracycline. Serum:broth ratios were bacterial species dependent for *A. pleuropneumoniae* and *P. multocida*; differences were evident after correction for binding to serum protein for all three indices of potency, indicating that correction for protein binding (whilst necessary) is not sufficient to account for matrix potency differences. Time–kill data demonstrated codependency of killing action for both *A. pleuropneumoniae* and *P. multocida* in both media. It is concluded that the *in vitro* potency of oxytetracycline is likely to be better indicated by serum than by broth MICs. Differences between broth and serum will have implications for optimization of dosage schedules. Future studies should integrate and model pharmacokinetic data with pharmacodynamic indices generated in serum to determine optimal dosing, for confirmation (or not) in clinical studies.

## Conflict of Interest

None of the authors of this article have a financial or personal relationship with other people or organization that could inappropriately influence or bias the content of the article other than Norbrook Laboratories Ltd. who cosponsored this work with BBSRC.

During the last 5 years, the authors' interests have included the following: P Lees (consultancy advice supplied to Bayer Animal health, Norbrook Laboratories Ltd. and Pfizer Animal Health) and S. Hobson (employee of Norbrook Laboratories Ltd.).
